# Residents’ Learning Experiences about Patients’ Social Difficulties in the Emergency Department: Qualitative Research

**DOI:** 10.31662/jmaj.2025-0202

**Published:** 2025-09-26

**Authors:** Junki Mizumoto, Hirohisa Fujikawa

**Affiliations:** 1 Center for General Medicine Education, School of Medicine, Keio University, Tokyo, Japan; 2 Department of Family Practice, Ehime Seikyo Hospital, Matsuyama, Japan; 3 Department of General Medicine, Juntendo University Faculty of Medicine, Tokyo, Japan; 4 Department of Medical Education Studies, International Research Center for Medical Education, Graduate School of Medicine, University of Tokyo, Tokyo, Japan

**Keywords:** complexity, social determinants of health, social emergency medicine, qualitative research

## Abstract

**Introduction:**

Emergency departments (EDs) are increasingly tasked with addressing the complex needs of patients whose daily lives are threatened by non-biomedical factors. To optimize learning about social determinants of health in EDs, it is important to better understand residents’ experiences and learning processes in this context.

**Methods:**

This qualitative study aimed to explore how residents with positive attitudes toward treating patients with social difficulties in the ED approach such cases and what they learn from these interactions. We selected a hospital where many ED patients present with complex social needs. The hospital is committed to non-discriminatory and equitable medical care and welfare, with educational policies that integrate patients’ social backgrounds into daily care. Physicians in postgraduate years 1-7 were recruited purposively. In-depth online interviews were conducted, and data were analyzed using thematic analysis (a framework approach).

**Results:**

A total of 13 physicians participated, with three main themes emerging: (i) healing care in the ED, (ii) positive learning experiences, and (iii) conflict and resolution. Participants described providing healing care in the ED, noting the challenges they faced and the learning opportunities they gained. They emphasized the importance of understanding and responding to patients’ needs while noting conflicts in the workplace.

**Conclusions:**

Novice physicians who have a positive attitude toward social problems in EDs approached patients with a non-judgmental attitude, provided healing-oriented care, and aimed to foster their professional development. They often experienced conflict that experts in health professions education need to address to better support residents.

## Introduction

The primary role of an emergency department (ED) is to provide life-saving care to patients with acute medical emergencies. However, EDs are increasingly being called upon to address the complex needs of patients whose daily lives are affected by non-biomedical factors, often referred to as social determinants of health (SDHs). Socioeconomic factors are associated with frequent ED visits ^(1), (2)^, and patients at higher social risk often present to the ED with non-urgent symptoms ^(3)^. In such cases, a purely biomedical approach may be insufficient to address the underlying issues. Because of its continuous accessibility and non-selective acceptance of various health problems, the ED often becomes a point of contact for socially marginalized individuals who lack essential resources to manage their daily lives ^(4)^. Indeed, many patients who present to the ED may urgently require social support ^(5), (6)^. In particular, EDs in community hospitals and clinics tend to encounter fewer patients requiring advanced life support and a greater number of patients who present with a broad spectrum of health and social concerns.

The ED can be viewed as a window into the community and its social inequities ^(7)^, serving as a valuable environment for learning clinical practices influenced by the patients’ SDHs ^(8), (9)^. As health care providers increasingly recognize the importance of addressing SDHs, research in social emergency medicine has gained prominence ^(10)^. However, a traditional emergency medicine education may lack a focus on SDHs and the patients’ social risks and needs ^(11)^. Clinical education has traditionally emphasized disease management and technical skills, with less attention to SDHs ^(12)^. In addition, social topics tend to have unclear learning objectives and vague criteria for assessing learners’ competence. As a result, residents may perceive these topics as undervalued and disconnected from clinical practice, potentially impeding the development of empathy, caregiving skills, and the capacity to advocate effectively for patients ^(13)^.

Previous studies suggest that SDH-focused education may improve emergency medicine residents’ knowledge of SDHs, increase their awareness of patients’ social needs, and enhance their approach to care ^(11), (14)^. It has also been suggested that medical students may better recognize the importance of SDHs through discussions with patients and team-based feedback ^(15)^. However, studies examining learners’ behaviors and experiences, as well as their interactions with patients and the clinical environment, are limited. There is also no consensus on the best methods for observing and evaluating SDH-related clinical learning that arises from the interaction between residents, patients, and environmental factors ^(16)^.

In Japan, pre-graduate competencies related to SDHs were incorporated into the Model Core Curriculum for Medical Education in Japan (revised in 2022) ^(17), (18)^. Studies have shown that medical students can deepen their conceptual understanding of SDHs through community engagement and observational learning ^(19)^. In postgraduate education, residents have been reported to develop the ability to anticipate underlying challenges in patients’ lives and revise overly idealized, uniform perceptions of patients through learning about SDHs ^(20)^. However, little is known about what residents in Japan learn and experience regarding SDHs in their clinical encounters, particularly, in the ED.

Existing literature indicates that acknowledging patients’ social backgrounds and contexts and delivering patient-centered care in the ED can improve patient outcomes ^(21)^. Given the limited opportunities for residents to learn and practice such care in this setting ^(21), (22)^, it is essential to examine their learning experiences in relation to SDHs. To optimize educational opportunities in the ED, where many patients with social problems seek care, it is crucial to better understand how residents learn and what they experience in this setting. To understand a developmental trajectory for providing high-quality care, this study qualitatively explores how residents with positive attitudes toward treating patients with social difficulties in the ED approach such patients, and what experiences and lessons they draw from these interactions.

## Materials and Methods

### Setting

The hospital where this study was conducted has approximately 400 beds, is located in a suburb of a large city in Japan, and receives approximately 1,100 local ED patients per month. The hospital is surrounded by a socially disadvantaged area, and many patients with different social needs come to the hospital. The hospital is committed to non-discriminatory and equal medical care and welfare, and one of its educational policies is to provide care based on the social determinants of patients. To ensure the anonymity of the participants, the name of the hospital is not mentioned.

This qualitative study was reported according to the Standards for Reporting Qualitative Research ^(23)^. Recruitment, interviews, and data analysis discussions in this study were conducted online because of geographic dispersion.

The epistemology of social constructivism was adopted. Constructivists recognize that individuals construct different understandings based on past experiences and knowledge. Social constructivism posits that learning is shaped by interplays between individuals and their environment, which includes other people, objects, and activities present. This dynamic is evident in learners’ engagement with real-world practice, especially when learners encounter conflicting ideas. The theory insists that knowledge is individually constructed, with learners actively involved in the learning process ^(24)^. This study aimed to reveal residents’ learning experiences gained by the interaction with patients and other surroundings in clinical settings, and it is assumed that participants develop their own understanding of how to interact with patients, other medical staff, and other stakeholders through their experiences in the ED.

### Reflexivity

The first author (J.M.) and the second author (H.F.) are primary care physicians and researchers in health professions education (HPE). Neither has worked as a physician in this hospital. Throughout the research process, we prioritized gaining a deeper understanding of participants’ experiences while remaining aware that their interpretations were shaped by their own backgrounds in patient care and professional development as primary care physicians.

J.M. conducts research and education on integrating SDHs into clinical care. In 2022, the hospital held a workshop on how to manage patients in complex and challenging social situations by reviewing patients with social problems in the ED, and J.M. participated as an instructor. In this workshop, J.M. and multi-professional members of the hospital discussed one illustrative case, and some residents (postgraduate years [PGYs] 1 or 2 at that time) participated. Through the workshop, J.M. found that the hospital welcomed many patients with socially complex needs, and many residents expressed positive attitudes toward addressing the patients’ social problems in the ED. This experience inspired this study. We believe that residents and attending physicians working in the hospital would be potential participants in this research question.

### Participants

Participants were recruited through purposive sampling. Participants should meet all inclusion criteria as follows: (i) physicians in PGYs 1 to 7 who were currently working or had previously worked in the hospital’s ED, (ii) individuals who self-identified as having a positive attitude toward caring for patients facing complex social challenges (self-reported criteria), and (iii) individuals who were deemed by the head of the ED to possess such attitudes (ED head’s subjective assessment). J.M., with the assistance of the head of the ED and the residency coordinator, contacted potential participants by email. All individuals who were invited agreed to participate and included in the study.

### Data collection

To reveal the participants’ experience of dealing with patients’ social difficulties in the ED, mainly, during their PGYs 1-2, J.M. conducted each in-depth interview, using a semi-structured interview guide (Appendix 1). Participants were asked to share their most memorable experiences related to patient encounters, the provision of care, and learning opportunities in the ED. This guide was initially developed based on the experience of J.M. and subsequently refined through several rounds of interviews. Physicians in PGYs 2-7 were interviewed from May to June 2024. In Japan, the fiscal year begins in April, so participants were just beginning their new PGYs. Then, physicians in PGY 1 were interviewed from October to November 2024 (approximately a half year after beginning their clinical practice). Participants in PGY 1 and PGY 2 (current residents) were asked to share their real-time experiences from the beginning of their training until the interview. Participants in PGY 3 or higher were asked to reflect on their experiences during PGY 1 and PGY 2 retrospectively, as well as their experiences supervising residents (PGY 1 and PGY 2 physicians) in the ED. The interviews were conducted online via Zoom because of geographic dispersion. Each interview lasted between 30 and 50 minutes, and the Zoom meeting rooms were password-protected. The recorded video was deleted immediately after the interviews, and only the recorded audio was kept for transcription.

### Data analysis

An inductive thematic analysis of the interviews was performed, using a framework approach ^(25)^. The framework approach is a recognized method within thematic analysis, offering a structured and systematic process for data interpretation. This approach usually involves seven steps: verbatim transcription, familiarization with the complete interview, initial coding, development of a working analytical framework, re-application of the framework to the entire data set, summarization of data into the framework, and data interpretation. J.M. and H.F. iteratively coded and discussed the data, merging their analyses at each step until a consensus was reached. In instances of disagreement, J.M. and H.F. deliberated collaboratively and reached a final agreement on the coding. Written notes documenting the analytic process using a framework approach facilitated the exchange of ideas between J.M. and H.F. and supported critical reflection on their own positionality and reflexivity. Finally, all participants received the coding by email, reviewed it, and made any necessary revisions (member checking). During this process, no external reviewer was involved.

### Ethical considerations

J.M. contacted potential participants by email, clarifying that participation was entirely voluntary and that declining to participate would not result in any adverse consequences. Participants submitted written informed consent, including permission for J.M. to preserve their audio-recorded comments until five years after the paper is published. Interviews were transcribed and anonymized by J.M. and subsequently shared with H.F. Each transcript was assigned a participant number for identification. All proper nouns and any potentially identifying expressions were anonymized to ensure confidentiality. Participants were offered an incentive of a JPY ¥2,000 (approximately USD $13) gift voucher. This study was approved by the Research Ethics Committee of the Ehime Seikyo Hospital (reference No. 51-2-2024-001). The committee determined that additional ethical approval at the study site was not required.

### Criteria for ensuring quality

To ensure the quality of our findings, we evaluated our research methods according to the quality criteria widely used in qualitative research, as outlined in the subsequent section ^(26)^.

#### (i) Credibility

We collected data triangulation by including not only current residents (PGYs 1-2). Participants in PGY 3 also reported their experiences as residents and those in PGYs 4-7 as attending physicians, thereby capturing diverse perspectives on residents’ experiences in the ED. Investigator triangulation was achieved through the involvement of J.M. and H.F. in the coding process, enabling continuous dialogue and iterative validation of the coding framework. However, J.M. and H.F. shared similar professional backgrounds and this may have constrained the breadth of this triangulation. Furthermore, member checking was conducted by soliciting feedback from participants to ensure the accuracy and trustworthiness of data interpretations.

#### (ii) Transferability

We provided rich and thick descriptions by incorporating extensive direct quotations from participants. The sampling strategy was clearly documented to allow readers to assess the applicability of findings to other contexts.

#### (iii) Dependability

Dependability was ensured by pursuing data saturation and continuing data collection until no new themes emerged. The process was guided by an iterative approach in which ongoing analysis informed subsequent data collection. Using the framework method, we repeatedly revisited the data, incorporating emerging insights throughout the analytic process.

#### (iv) Confirmability

A framework analysis also contributed to transparent and detailed documentation about each step of the analysis, which underwent continuous review. Grounded in a social constructivist paradigm, we engaged in reflexive practices, individually acknowledging and examining their roles, perspectives, and potential influences on the research process.

## Results

Thirteen residents and physicians participated. All participants had PGYs 1-2 residency experience at the hospital, except for participant 7. Participant 7 experienced residency for PGYs 1-2 in a smaller hospital outside the area and has worked in the hospital since PGY 3. He was invited to share his perspectives based on his experience supervising residents in the ED. His distinctive background, which differs somewhat from that of other participants, enriched and deepened our overall understanding. The interview guide remained unchanged throughout. The details of the participants’ demographics are shown in Table 1. Some PGYs 2 or 3 participants also attended the workshop held in 2022, although we did not know the details of their attendance. Member checking confirmed agreement with the analysis.

**Table 1.** Participants’ Demographics.

No.	Gender	Age	Postgraduate year (PGY)	Specialty (PGY 3 or higher)
1	Male	27	3	Pediatrics
2	Male	26	2	
3	Female	27	3	Obstetrics and gynecology
4	Female	25	2	
5	Male	34	2	
6	Male	30	7	Internal medicine
7	Male	35	6	Internal medicine
8	Male	28	2	
9	Male	29	4	Internal medicine
10	Male	28	3	Internal medicine
11	Male	26	2	
12	Male	26	1	
13	Male	26	1	

Participants shared their experiences of practicing healing care in the ED, acknowledging the challenges and the learning opportunities this presented. They emphasized the importance of understanding and responding to patients’ needs, even when these needs extend beyond immediate biomedical emergencies. Participants also reported experiencing conflict in their workplace.

### Healing care in the ED: “I’m all ears!”

#### Focus on patient needs

Participants emphasized the importance of respecting the patient’s experience of illness and focusing on their needs, regardless of the medical diagnosis. Participants were guided by their supervisors to ensure that each ED visit was meaningful for patients by either alleviating their distress or, at the very least, providing them with information on how to address the issues that prompted their visit.

*What the patient feels, such as pain and suffering, is an absolute fact for the patient and we cannot deny it. Even if there is no biomedical condition, for the patient, it is such an emergency that an ambulance has to be called. (Participant 5: PGY 2)*

Participants found that their careful listening to patients’ narratives encouraged patients to open up about the social and personal challenges they faced. Many participants noted the importance of simply sitting at the bedside and listening to the patient’s narrative.

*Only after a series of complaints do such patients shift their modes to talk in detail about their lives. The patient told us that he was sick because of heavy drinking and no job, which developed a negative cycle: he had enough time to drink. (Participant 2: PGY 2)*

Although recognizing the importance of assessing whether a patient’s distress was a biomedical emergency, participants focused on how to resolve the patient’s distress without making a judgment about whether the patient was eligible for care in the ED.

*The social problems that patients face are often too much of a burden for the patients to solve alone. If I can find a possible solution, I would like to present it. [...] Biomedical remedies do not always send patients home with a smile on their faces. (Participant 9: PGY 4)*

Participant 5 (PGY 2) explained that for patients without a medical emergency, listening attentively to their narratives―understanding what was troubling them and why they chose to call an ambulance at that moment―enabled physicians to uncover and reflect on the underlying reasons for the visit, thereby alleviating their distress. The participant emphasized that physicians should address patients’ concerns, regardless of underlying factors.

*We should keep on addressing every difficulty faced by the patient in front of us. (Participant 5: PGY 2)*

#### The healing power of listening

Participants recognized that a physician’s act of listening to patients can be inherently therapeutic. Participants consistently experienced the therapeutic impact of attentive listening as they engaged in the ongoing provision of earnest and compassionate patient care.

*The fact that a doctor really listens to a patient must have a healing effect in itself [...]. The emergency department is often the only place where patients can have their narratives and current distress heard by health care professionals, with the assurance that their lives are entrusted and safeguarded. (Participant 6: PGY 7)*

Even in the absence of direct medical intervention, patients often left the ED feeling better, attributing this improvement to the attentive and sincere care they received.

*The physician’s sincerity helps alleviate the patient’s distress [...]. Some patients have experienced harsh treatment in the past, which I can tell during consultations. By seeing them sincerely, they often express their gratitude for the care I provide, and they eventually leave with a smile, saying their pain is gone. (Participant 11: PGY 2)*

Some participants felt that they provided patients with a reliable social connection.

*I think one of the reasons patients come to see a doctor is to have someone to listen to when they feel isolated. It’s important to let them know that there are people who will listen, including me. (Participant 2: PGY 2)*

Participant 3 reported an encounter with a patient who frequently presented to the ED with complaints of chronic pain. The patient consistently expressed severe discomfort, at times, in a coercive and emotionally charged manner, conveying significant anxiety and tension. Through engagement with this patient, the participant came to recognize the therapeutic value of listening.

*I experienced that the patient, who usually leaves home after receiving a pack of Acelio^Ⓡ^ (paracetamol), felt much relieved after a brief conversation with the ER staff. Maybe what he really needed was a tie with others. (Participant 3: PGY 3)*

#### Care facilitated by being a resident

Participants saw the healing care they provided as something made possible by their position as residents, with more flexibility in the ED. They consciously used this role to provide more attentive care.

*In an environment where the attending is busy managing the whole department, it becomes my responsibility to listen attentively to the patient [...]. Knowing that I am primarily responsible for this patient, I can and should take the time to listen carefully. (Participant 4: PGY 2)*

### Positive learning experiences: “Come to our emergency room!”

#### Acceptance despite being overwhelmed by the patients’ narratives

Participants often felt overwhelmed by the sheer volume and intensity of the patients’ stories, yet they continued to listen and respond with empathy.

*As soon as the patient saw me, she said that she couldn’t afford the charge [...]. I had always been aware that some patients were more concerned about financial issues than their health when being admitted to the hospital, but this encounter made me realize that such cases really do exist. (Participant 4: PGY 2)*

*I asked the patient why he drank so much. He replied that he had hallucinations, maybe due to his adverse experience, and that he was trying to escape from them. I was really astounded and shocked. The hallucinations ruined his whole life. (Participant 5: PGY 2)*

Through these experiences, participants learned the importance of remaining open and honest with their patients.

*Even if I develop negative feelings, responding to the patient with sincerity can have a positive effect on both the patient and myself [...]. I make every effort to manage my negative feelings by responding with genuine care for the patient. (Participant 11: PGY 2)*

#### Desire for quality care

Participants learned that understanding the social determinants can reveal the underlying causes of the patients’ problems and enable more effective care.

*It’s too late to find out after several tests that the patient simply can’t afford food. If I know the patient’s social background earlier, I can intervene before their conditions get worse and prevent frequent visits to the emergency department. (Participant 8: PGY 2)*

*If I ignore where the patient’s anxiety is coming from and send the patient home just because there doesn’t seem to be a biomedical problem, it’s substandard care. (Participant 1: PGY 3)*

Addressing the patients’ social challenges became a professional goal for some participants.

*I believe that a truly exceptional physician should be able to address patients’ social problems. (Participant 9: PGY 4)*

*If we can treat patients properly here in this department, where we face many complex social challenges, we can always treat patients in other settings. (Participant 12: PGY 1)*

#### The experience of getting by

Participants recognized that physicians could help address social problems by coordinating with interprofessional teams and community resources.

*I collected all the patient information in the hopes that consultation with experts would provide a solution. Some stories were overwhelming, but I was able to gather the necessary details [...]. I know I could choose to do nothing and ignore these cases, but, now, I understand that our extended team is ready to help. If I introduce the patient to the team, they may be able to find a solution. I’ve seen it happen several times. (Participant 6: PGY 7)*

### Conflict and resolution: “I’m between Scylla and Charybdis!”

#### Conflict with biomedical assessment and management

Participants sometimes encountered conflicts between addressing patients’ social issues and fulfilling their biomedical responsibilities, particularly, in emergency settings where time and resources are limited.

*If there’s a critically ill patient in the bed next to the patient I’m talking to, I can’t spend enough time understanding their social background. (Participant 10: PGY 3)*

Acute care wards required strict criteria for admission, which further complicated the situation for patients with social problems.

*As long as our hospital provides acute care, we can’t admit patients whose main problems are social [...]. It was a challenge when I was told that these patients couldn’t be admitted with their conditions. (Participant 7: PGY 6)*

To mitigate this conflict, participants emphasized the need for an environment where learners could focus on patients holistically.

*In order for residents to spend enough time listening to their patients, ED staff need to understand the value of the ED. We need to recognize that the ED serves patients with social problems and creates an environment where residents can listen carefully. (Participant 6: PGY 7)*

#### Conflict with supervisors and other staff

Participants also reported tensions with supervisors and other staff who were frustrated by the time spent dealing with patients’ social problems during busy shifts.

*I dread being on night duty with an attending who discourages me from talking to my patients. (Participant 1: PGY 3)*

*“Why don’t you just send that noisy patient home?” [...] When I hear things like that from supervisors, I know they’re tired, especially during the hardest parts of the shift. (Participant 5: PGY 2)*

*I often feel pressured by supervisors and nursing staff to minimize the time spent with each patient. Under such constraints, it becomes challenging to explore the patient’s social background in depth. (Participant 13: PGY 1)*

In some cases, participants negotiated with their supervisors to resolve the conflict.

*I explain to my bosses that I understand there’s no urgency, and I assure them that I’ll cut the conversation short if things get busy. Then, they usually say, ‘That’s fine, do what you want for now.’ (Participant 5: PGY 2)*

Conversely, participants recognized the positive impact of supportive supervisors on their professional development.

*I’ve worked with supervisors who take it for granted to consider the social background of the patients. They’ve taught me that if you don’t intervene in that, you can’t fully address the illness. (Participant 11: PGY 2)*

## Discussion

This study recruited residents who showed a positive attitude toward managing complex and challenging social situations and qualitatively explored their experiences and learning in the ED. Rather than limiting themselves to a strict biomedical approach, these physicians aimed to alleviate patient distress, reflecting a broader perspective on patient care in the ED. They saw their role not as gatekeepers but as compassionate providers committed to solving the patients’ problems holistically. Previous research suggests that maintaining a non-judgmental attitude toward frequent ED users can improve care management ^(27)^. In addition, the participants reported the healing effect of listening attentively to the patients’ narratives. This is consistent with Balint’s concept of “doctor as a drug ^(28)^,” where the doctor-patient relationship functions as a “mutual investment bank,” growing in value through mutual trust and commitment. Acceptance of marginalized patients can provide a therapeutic benefit in itself ^(29)^. Although traditional approaches to addressing SDHs in the ED focus on identifying and intervening in specific patient problems ^(30), (31), (32)^, the findings of this study suggest the need to consider more holistic approaches that prioritize patient healing in the ED setting.

Participants were often overwhelmed by the challenges faced by their patients, yet their experiences of extended team interventions empowered them to address these difficulties proactively. EDs typically operate with limited resources to address health-related social needs ^(32)^, and interprofessional collaboration is essential. For example, specific areas of social work practice have been identified within EDs ^(33)^. This study suggests that residents can play a valuable role by using their flexibility to gather comprehensive patient information and provide healing-oriented care. Recognizing the unique advantages of their role as residents may enhance their contribution to effective care.

This study offers implications for ED practices and valuable insights for experts in HPE on postgraduate education to address social problems in ED. First, it is important to recognize and work to resolve conflicts in an ED setting. As the clinical environment becomes more demanding and complex, supervisors should foster an environment that allows residents to spend meaningful time at the bedside with patients. Second, clinical supervisors should highlight available resources and social care services in the ED and ensure that they are well-developed. Many residents report discomfort when discussing social needs with patients and their families, and targeted social care training could improve their comfort and effectiveness in these conversations ^(34)^. Improved resources are also critical to preventing burnout among professionals ^(35), (36)^. Third, it is recommended that competence in addressing the patients’ social challenges be seen as an integral part of professional development, as highlighted by the participants in this study. Learning about SDHs can help residents see their role as encompassing not only biomedical issues but also the social contexts that affect patients’ health ^(37)^. Supporting residents in setting goals around these competencies can create a more dynamic environment in which residents can develop their skills to meet the patients’ needs ^(38)^.

This study has some limitations. First, the number of participants was relatively small. However, to ensure collection of enough data, data collection and analysis were conducted simultaneously, and recruitment was stopped after no new themes emerged from the participants’ input ^(39)^. The relatively rapid saturation may be because of the limited diversity of the participants’ backgrounds. We purposively selected a hospital recognized for its commitment to HPE in social emergency medicine in Japan. However, recruiting participants in a single institution may limit the generalizability of the findings. Future research in diverse settings may provide broader insights. Second, this study focused on the residents’ perspectives and did not include the perspectives of supervisors, patients, or non-medical professionals. Further studies designed to capture the perspectives of these groups, particularly, from marginalized populations, would provide a more comprehensive understanding of the dynamics of care. Recruitment of marginalized populations requires careful consideration to ensure ethical participation ^(40), (41)^. Third, conducting interviews online may constrain the richness of nonverbal communication and the level of rapport and trust that can typically be fostered in face-to-face interactions. Fourth, we included participants in PGYs 6-7 because they were expected to offer insights from instructional and early-career perspectives. However, their accounts of initial learning experiences may be subject to retrospective reinterpretation.

This study was conducted at a single hospital primarily providing secondary care, and participants were recruited based on their positive attitudes toward addressing the patients’ social challenges. When interpreting and applying the findings to other contexts, it is important to consider the specific characteristics of one’s own clinical and educational environments.

In conclusion, residents who were positive about the social challenges in EDs approached patients with a non-judgmental attitude, using their role as residents to provide compassionate, healing-oriented care. Although they often felt overwhelmed by the complexity of the patients’ narratives, these experiences enhanced their professional growth in an ED setting. However, they experienced conflict when addressing the patients’ social backgrounds―an area that experts in HPE need to address to better support residents. Future research should include a variety of perspectives, particularly, those of patients, to gain a more holistic view.

## Article Information

### Author Contributions

Junki Mizumoto contributed to the conceptualization, methodology, software, formal analysis, investigation, data curation, writing - original draft, visualization, and project administration. Hirohisa Fujikawa contributed to the methodology, validation, formal analysis, and writing - review & editing.

### Conflicts of Interest

None

### Ethical Approval

All participants signed an informed consent statement prior to participation in the study. The study received approval from the Research Ethics Committee of the Ehime Seikyo Hospital (reference No. 51-2-2024-001).

## Supplement

Supplementary Material

## References

[1] Davis CI, Montgomery AE, Dichter ME, et al. Social determinants and emergency department utilization: findings from the Veterans Health Administration. Am J Emerg Med. 2020;38(9):1904-9. 32739860 10.1016/j.ajem.2020.05.078

[2] Hellmann R, Feral-Pierssens AL, Michault A, et al. The analysis of the geographical distribution of emergency departments’ frequent users: a tool to prioritize public health policies? BMC Public Health. 2021;21(1):1689. 34530780 10.1186/s12889-021-11682-z PMC8447576

[3] McCarthy ML, Zheng Z, Wilder ME, et al. The influence of social determinants of health on emergency departments visits in a Medicaid sample. Ann Emerg Med. 2021;77(5):511-22. 33715829 10.1016/j.annemergmed.2020.11.010 PMC9228973

[4] Kangovi S, Barg FK, Carter T, et al. Understanding why patients of low socioeconomic status prefer hospitals over ambulatory care. Health Aff (Millwood). 2013;32(7):1196-203. 23836734 10.1377/hlthaff.2012.0825

[5] Behr JG, Diaz R. Emergency department frequent utilization for non-emergent presentments: results from a regional urban trauma center study. PLoS One. 2016;11(1):e0147116. 26784515 10.1371/journal.pone.0147116 PMC4718591

[6] Doran KM, Kunzler NM, Mijanovich T, et al. Homelessness and other social determinants of health among emergency department patients. J Soc Distress Homeless. 2016;25(2):71-7.

[7] Anderson ES, Lippert S, Newberry J, et al. Addressing social determinants of health from the emergency department through social emergency medicine. West J Emerg Med. 2016;17(4):487-9. 27429706 10.5811/westjem.2016.5.30240 PMC4944812

[8] Bernstein SL, Haukoos JS. Public health, prevention, and emergency medicine: a critical juxtaposition. Acad Emerg Med. 2008;15(2):190-3. 18275450 10.1111/j.1553-2712.2008.00055.x

[9] Anderson ES, Hsieh D, Alter HJ. Social emergency medicine: embracing the dual role of the emergency department in acute care and population health. Ann Emerg Med. 2016;68(1):21-5. 26921967 10.1016/j.annemergmed.2016.01.005

[10] Shah R, Della Porta A, Leung S, et al. A scoping review of current social emergency medicine research. West J Emerg Med. 2021;22(6):1360-8. 34787563 10.5811/westjem.2021.4.51518 PMC8597693

[11] Shufflebarger EF, Willett M, Sontheimer SY, et al. Feasibility of a multifaceted social emergency medicine curriculum for emergency medicine residents. West J Emerg Med. 2023;24(3):495-501. 37278805 10.5811/westjem.59009 PMC10284534

[12] Hoskin ER, Johnsen DC, Saksena Y, et al. Dental Educators’ Perceptions of Educational Learning Domains. J Dent Educ Dent. 2019;83(1):79-87. 10.21815/JDE.019.010 30600253

[13] Endres K, Burm S, Weiman D, et al. Navigating the uncertainty of health advocacy teaching and evaluation from the trainee’s perspective. Med Teach. 2022;44(1):79-86. 34579618 10.1080/0142159X.2021.1967905

[14] Ali S, Saleem SG, Khatri A, et al. “To teach or not to teach- that is the question” The educational and clinical impact of introducing an outcome based, modular curriculum in Social Emergency Medicine (SEM) at a private tertiary care center in Karachi, Pakistan. BMC Med Educ. 2023;23(1):429. 37301880 10.1186/s12909-023-04385-z PMC10257367

[15] Moffett SE, Shahidi H, Sule H, et al. Social determinants of health curriculum integrated into a Core emergency medicine clerkship. MedEdportal. 2019;15:10789. 30800989 10.15766/mep_2374-8265.10789 PMC6354789

[16] Salhi BA, Zeidan A, Stehman CR, et al. Structural competency in emergency medical education: a scoping review and operational framework. AEM Educ Train. 2022;6(suppl 1):S13-22. 35783075 10.1002/aet2.10754 PMC9222890

[17] The Model Core Curriculum for Medical Education in Japan [Internet]. Japanese Core Curriculum. 2022 [cited 2025 Aug 1]. Available from: https://core-curriculum.jp/en

[18] Fujikawa H, Ando T, Endo A, et al. Competencies related to generalism for Japanese medical undergraduates: essential skills for comprehensive care. Med Teach. 2024;46(suppl 1):S21-30. 39545503 10.1080/0142159X.2024.2385133

[19] Haruta J, Takayashiki A, Ozone S, et al. How do medical students learn about SDH in the community? A qualitative study with a realist approach. Med Teach. 2022;44(10):1165-72. 35583394 10.1080/0142159X.2022.2072282

[20] Mizumoto J, Fujikawa H, Izumiya M, et al. Residents’ learning and behavior about tool-guided clinical assessment of social determinants of health. J Gen Fam Med. 2024;25(2):87-94. 38481747 10.1002/jgf2.674 PMC10927908

[21] Walsh A, Bodaghkhani E, Etchegary H, et al. Patient-centered care in the emergency department: a systematic review and meta-ethnographic synthesis. Int J Emerg Med. 2022;15(1):36. 35953783 10.1186/s12245-022-00438-0 PMC9367087

[22] Litwin S, Vaillancourt S, Labelle FK, et al. Recommendations for patient-centered emergency care. CJEM. 2024;26(8):513-9. 38904747 10.1007/s43678-024-00706-3

[23] O’Brien BC, Harris IB, Beckman TJ, et al. Standards for reporting qualitative research: a synthesis of recommendations. Acad Med. 2014;89(9):1245-51. 24979285 10.1097/ACM.0000000000000388

[24] Thomas A, Menon A, Boruff J, et al. Applications of social constructivist learning theories in knowledge translation for healthcare professionals: a scoping review. Implement Sci. 2014;9(1):54. 24885925 10.1186/1748-5908-9-54 PMC4040365

[25] Gale NK, Heath G, Cameron E, et al. Using the framework method for the analysis of qualitative data in multi-disciplinary health research. BMC Med Res Methodol. 2013;13(1):117. 24047204 10.1186/1471-2288-13-117 PMC3848812

[26] Frambach JM, van der Vleuten CP, Durning SJ. AM last page. Quality criteria in qualitative and quantitative research. Acad Med. 2013;88(4):552. 23531762 10.1097/ACM.0b013e31828abf7f

[27] Schaad L, Graells M, Kasztura M, et al. Perspectives of frequent users of emergency departments on a case management intervention: a qualitative study. Inquiry. 2023;60:469580231159745. 36927138 10.1177/00469580231159745 PMC10026145

[28] Balint J. The doctor, his patient and the illness. 2nd ed. New York: Churchill Livingstone; 2000. 416 p.

[29] Mercer SW, Higgins M, Bikker AM, et al. General practitioners’ empathy and health outcomes: a prospective observational study of consultations in areas of high and low deprivation. Ann Fam Med. 2016;14(2):117-24. 26951586 10.1370/afm.1910 PMC4781514

[30] Abiri A, Evans DD, Hamilton JB. Strategies to integrate the practice of social emergency medicine into routine Patient Care. Adv Emerg Nurs J. 2022;44(2):78-83. 35476683 10.1097/TME.0000000000000409

[31] Murray E, Roosevelt GE, Vogel JA. Screening for health-related social needs in the emergency department: adaptability and fidelity during the COVID-19 pandemic. Am J Emerg Med. 2022;54:323.e1-4. 10.1016/j.ajem.2021.09.071 PMC8492605 34654599

[32] Samuels-Kalow ME, Boggs KM, Cash RE, et al. Screening for health-related social needs of emergency department patients. Ann Emerg Med. 2021;77(1):62-8. 33160720 10.1016/j.annemergmed.2020.08.010 PMC7755764

[33] Bell J, Davies B, Walsh C, et al. The role and potential of social worker involvement in hospital emergency departments: A practice-based scoping review. Int J Soc Work. 2018;5(2):79.

[34] Assaf RR, Barber Doucet H, Assaf RD, et al. Social care practices and perspectives among U.S. pediatric emergency medicine fellowship programs. AEM Educ Train. 2022;6(2):e10737. 35493290 10.1002/aet2.10737 PMC9045575

[35] De Marchis E, Knox M, Hessler D, et al. Physician burnout and higher clinic capacity to address patients’ social needs. J Am Board Fam Med. 2019;32(1):69-78. 30610144 10.3122/jabfm.2019.01.180104

[36] Whitebird RR, Solberg LI, Crain AL, et al. Clinician burnout and satisfaction with resources in caring for complex patients. Gen Hosp Psychiatry. 2017;44:91-5. 27432586 10.1016/j.genhosppsych.2016.03.004

[37] Mizumoto J, Son D, Izumiya M, et al. Experience of residents learning about social determinants of health and an assessment tool: mixed-methods research. J Gen Fam Med. 2022;23(5):319-26. 36093216 10.1002/jgf2.559 PMC9444009

[38] Mizumoto J, Mitsuyama T, Kondo S, et al. Defining the observable processes of patient care related to social determinants of health. Med Educ. 2023;57(1):57-65. 35953461 10.1111/medu.14915

[39] Guest G, Namey E, Chen M. A simple method to assess and report thematic saturation in qualitative research. PLoS One. 2020;15(5):e0232076. 32369511 10.1371/journal.pone.0232076 PMC7200005

[40] Qi D, Abri K, Mukherjee MR, et al. Health impact of street sweeps from the perspective of healthcare providers. J Gen Intern Med. 2022;37(14):3707-14. 35296981 10.1007/s11606-022-07471-y PMC9585118

[41] George S, Duran N, Norris K. A systematic review of barriers and facilitators to minority research participation among African Americans, Latinos, Asian Americans, and Pacific Islanders. Am J Public Health. 2014;104(2):e16-31. 10.2105/AJPH.2013.301706 PMC3935672 24328648

