# Supplementary material for: Residents’ Learning Experiences about Patients’ Social Difficulties in the Emergency Department: Qualitative Research
Source: JMA J. 2025 Sep 26;8(4):1359–67. doi: 10.31662/jmaj.2025-0202 (PMC12598151; doi:10.31662/jmaj.2025-0202)
Supplement: Supplementary Material [file 2433-3298-8-4-1359-s001.pdf]

Appendix 1. Interview Guide.

- Have you dealt with patients who visit the emergency department primarily for social difficulties?
- Please describe your impressive cases.
- What measures did you take to deal with the situation and why?
- What have you learned from dealing with such patients?
- Why are you able to deal with patients with social difficulties in a positive way?
